# A taxonomic study of the genus *Panesthia* (Blattodea, Blaberidae, Panesthiinae) from China with descriptions of one new species, one new subspecies and the male of *Panesthia antennata*

**DOI:** 10.3897/zookeys.466.8111

**Published:** 2014-12-19

**Authors:** Xiudan Wang, Zongqing Wang, Yanli Che

**Affiliations:** 1 Institute of Entomology, College of Plant Protection, Southwest University, Beibei, Chongqing 400716, China

**Keywords:** Wing polymorphism, brachypterism, illustration, key, *Salganea*

## Abstract

One new species *Panesthia guizhouensis* **sp. n.** and one new subspecies *Panesthia stellata concava* **ssp. n.** are described and illustrated. The male of *Panesthia antennata* Brunner von Wattenwyl, 1893 and its brachypterous form are described and illustrated for the first time. *Panesthia strelkovi* Bey-Bienko, 1969 is redescribed and illustrated. Three known species, *Panesthia birmanica* Brunner von Wattenwyl, 1893, *Panesthia sinuata* Saussure, 1839 and *Panesthia angustipennis cognata* Bey-Bienko, 1969 are illustrated. In addition, a key to all species of the genus *Panesthia* from China is presented.

## Introduction

The wood-feeding cockroach genus *Panesthia* was established by Serville (1831), belonging to the subfamily Panesthiinae of the family Blaberidae. Brunner von Wattenwyl (1893) presented 16 species and Saussure (1895) recorded 33 species in this genus. Bey-Bienko (1969) described three species of this genus from China. More recently, Roth (1977, 1979) recognized 55 species and nine subspecies of *Panesthia* worldwide, of which 15 species and two subspecies were reported for the first time. In this latter work, Roth also stated that *Panesthia angustipennis spadica* by Bey-Bienko (1950) from mainland China should be *Panesthia angustipennis cognata*. From then on, no new valid taxon in this genus was published. Asahina (1988) established the subspecies *Panesthia angustipennis yayeyamensis*, which was split from the subspecies *Panesthia angustipennis spadica*; but Maekawa et al. (1999) disagreed with his view based on molecular data. Feng and Woo (1990) reported two species from China, i.e., *Panesthia concinna* Feng & Woo, 1990 and *Panesthia guangxiensis* Feng & Woo, 1990; the former had been synonymized with *Salganea taiwanensis* Roth, 1979, while the latter was transferred to *Salganea* (Wang et al., 2014). At the same time, they also recorded *Panesthia birmanica*, *Panesthia sinuata* and *Panesthia stellata* as distributed in China. Up to now, there were 55 species and nine subspecies of *Panesthia* reported worldwide, including eight species and two subspecies from China.

In this paper, we report one new species and one new subspecies, and also provide a key including nine species and three subspecies of the *Panesthia* from China. We also take this opportunity to report the male and the brachypterous form of *Panesthia antennata* for the first time.

## Materials and methods

The terminology of the head, body and male genitalia used in this paper mainly follows Roth (1977, 1979, 2003). Measurements are based on materials examined. Measurement of body length is without the tegmen. The genital segments of the examined specimens were macerated in 10% NaOH and observed in glycerin jelly using a Motic K400 stereomicroscope. All drawings were made with the aid of a Motic K400 stereomicroscope. Photographs of the specimens were made using a Canon 50D plus a Canon EF 100mm f/2.8L IS USM Macro lens with the aid of Helicon Focus software. We considered adults and nymphs collected from the same colony with similar external characters to be one species. Nymphs were identified mainly based on markings on the mesonotum and metanotum as well as their holes in terga, lateral margin of terga and hind margin of supra-anal fig. Type specimens are deposited in the Institute of Entomology, Southwest University, Beibei, Chongqing, China (SWU) and the Museum of Hebei University, Baoding, Hebei Province, China (HBU). We also borrowed specimens from the Museum of Southwest Forestry University, Kunming, Yunnan Province, China (SWFU) and Dali University, Dali, Yunnan Province, China (DLU) as indicated.

## Taxonomy

### Family Blaberidae Brunner von Wattenwyl, 1865

#### Subfamily Panesthiinae Kirby, 1904

##### Genus Panesthia

Taxon classificationAnimaliaBlattodeaBlaberidae

Serville, 1831

Panesthia Serville, 1831: 38; Princis 1965: 309; Roth 1977: 12, 1979: 3. Type species: *Panesthia angustipennis* (Illiger, 1801).
Proterodia Costa, 1866: 5. Type species: *Proterodia punctatissima* Costa, 1866. Synonymized by Princis 1965: 309.
Dicellonotus Butler, 1882: 387. Type species: *Dicellonotus lucanoides* Butler, 1882. Synonymized by Roth 1977: 12.

###### Diagnosis

(**mainly following Roth 1977, 1979**). Coloration dark reddish brown or black. Size ranging from 15 mm to over 50 mm. Body strongly sclerotized with a coarse surface, densely covered with punctations. Vertex foveolar or not, slightly exposed. Pronotum transversal ovate, anterior margin slightly convex, with a variable excision in the midline, or entire. If excised, the corners of the concavity protruding or not. Lateral margins of pronotum arched and the hind margin almost straight or slightly concave. The surface of the pronotum granular on variably depressed anterior half with a pair of oblique grooves and often with two disc tubercles on the posteriorly punctate half. Tegmina and wings unicoloured or not, fully developed (sometimes mutilated terminally or only leaving the basal portion of the tegmina and wings), or reduced, or tegmina reduced but wings absent, or both tegmina and wings absent. The tarsi of legs with five segments, pulvilli are present on segments 1–4. The hind metatarsus is shorter than the remaining segments combined. Claws symmetrical, without arolia. Abdominal terga with punctate surface, and the hind margins without spines, tubercles or teeth.

Anterolateral corners of terga rarely with holes and without setae, or just tergum six (*T6*) and tergum seven (*T7*) with holes. Lateral margins of *T6* smooth, and laterocaudal angles not produced, or with a spine and directed caudally. Lateral margins of *T7* straight and not crenulate, laterocaudal angles sometimes produced and usually directed caudally. Lateral margins of sternite seven (*S7*) with a feeble and short ridge or without ridge. In the male, the hind margin of the last sternite is truncate or concave, and the subgenital fig is slightly exposed. In the female, the hind margin of the last sternite is convex and rounded. Both sexes are without styli. Supra-anal fig punctate, with uneven or rounded hind margin, and cerci are short and broad basally. Paraprocts are asymmetrical, the left one in ventral view with a finger-like projection lacking in the right one. Anterior margin and lateral margins of subgenital fig concave and the hind margin is rounded. Four genital phallomeres as follow: first sclerite of the left phallomere (*L1*) figd; second ventromedial sclerite of left phallomere (*L2vm*) rod-like; second dorsal sclerite of the left phallomere (*L2d*) variable; second sclerite of the right phallomere (*R2*) well developed or reduced, if developed, it is often hook-like and curved to right side in dorsal view.

###### Remarks.

The genus *Panesthia* is recognized by both *T6* and *T7* having smooth lateral margins, their hind margins without tubercles; the hind angles of *T7* spine-like, but *T6* not. Some species in this genus may have individuals with mixed characters resembling the genus *Salganea* Stål, 1877, *Ancaudellia* Shaw, 1925 or *Miopanesthia* Saussure, 1895 (Roth 1982: 71). The first two genera can be distinguished from *Panesthia* by the anterolateral angles of terga usually with holes or grooves with associated setae, but species of *Panesthia* often lack holes, or if with holes in *T6* and *T7*, the holes without setae. The last genus *Miopanesthia*, has a hind metatarsus that is usually close to or longer than the combined length of the remaining tarsal segments; however, the hind metatarsus is shorter than the remaining segments in *Panesthia*.

###### Distribution.

Oriental Region, Australian Region, a few locations in the Palaearctic Region (China, Japan).

###### Key to species of *Panesthia* from China

**Table:** 

1	Tegmina and wings absent	***Panesthia larvata***
–	Tegmina and wings present, or tegmina present and wings absent	**2**
2	Tegmina reduced (Fig. 17) and wings absent	***Panesthia strelkovi***
–	Tegmina and wings well developed or reduced	**3**
3	Tegmina with more than one colour in the form of spots	***Panesthia transversa***
–	Tegmina unicoloured without spots	**4**
4	Laterocaudal angles of *T6* acute and posteriorly directed	***Panesthia birmanica***
–	Laterocaudal angles of *T6* not acute	**5**
5	Anterior margin of pronotum broadly excavated and with a projection mesally in male, slightly concave and without projection in female	**6**
–	Anterior margin of pronotum slightly concave and without middle projection in both sexes	**8**
6	Body length < 30 mm, anteroventral margin of front femur with 0–1 spine	***Panesthia sinuata***
–	Body length > 30 mm, anteroventral margin of front femur with two spines or more (*Panesthia angustipennis* complex)	**7**
7	Hind margin of supra-anal fig entire dorsally or weakly undulate; median phallomere *L2d* elongate, tapering to a round apex (Roth 1979: Figs 19J, 20C–G)	***Panesthia angustipennis spadica***
–	Hind margin of supra-anal fig undulate dorsally; median phallomere *L2d* short with variable apex (Fig. 92; Roth 1979: Figs 21H, 22E, 23A–X)	***Panesthia angustipennis cognata***
8	Hind margin of supra-anal fig slightly crenate in ventral view (Fig. 89) and entire in dorsal view	***Panesthia guizhouensis* sp. n.**
–	Hind margin of supra-anal fig crenulate in ventral and dorsal view	**9**
9	Vertex with a foveola in dorsal view (Fig. 27), and teeth on supra-anal fig with smooth border (Fig. 32)	***Panesthia antennata***
–	Vertex without foveola (Fig. 39), and teeth on supra-anal fig with uneven border (Fig. 44)	***Panesthia stellata concava* ssp. n.**

**Figures 1–16.**
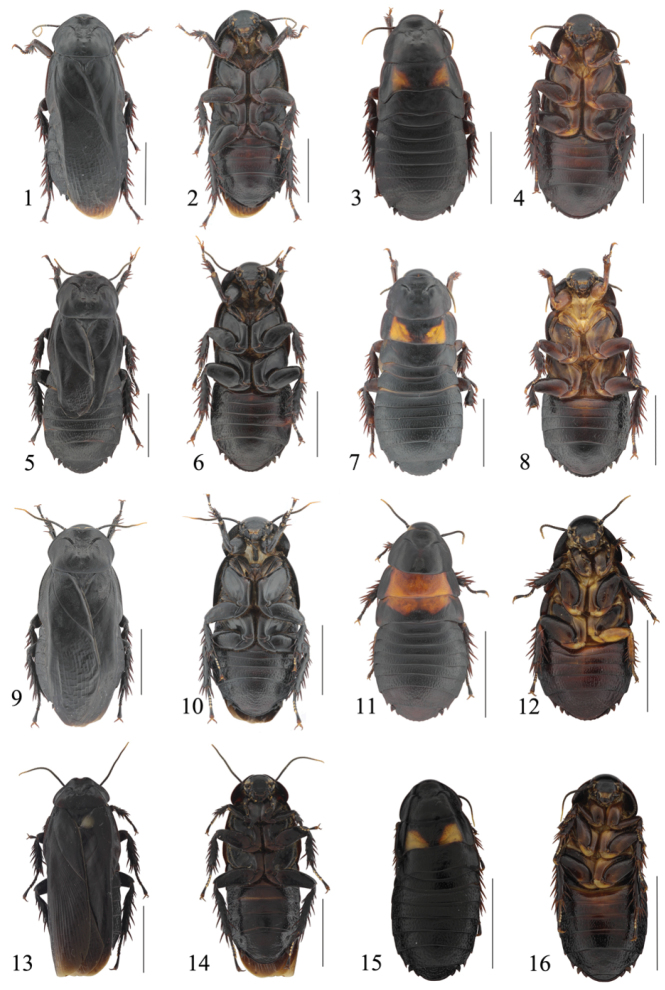
**1–2** *Panesthia antennata* Brunner von Wattenwyl, 1893, male: **1** dorsal view **2** ventral view **3–4** *Panesthia antennata* Brunner von Wattenwyl, 1893, nymph: **3** dorsal view **4** ventral view **5–6** brachypterous form of *Panesthia antennata* Brunner von Wattenwyl, 1893, male: **5** dorsal view **6** ventral view **7–8** brachypterous form of *Panesthia antennata* Brunner von Wattenwyl, 1893, nymph: **7** dorsal view **8** ventral view **9–10** *Panesthia stellata concava* ssp. n., male: **9** holotype, dorsal view **10** same, ventral view **11–12** *Panesthia stellata concava* ssp. n., nymph: **11** paratype, dorsal view **12** same, ventral view **13–14** *Panesthia guizhouensis* sp. n., male: **13** holotype, dorsal view **14** same, ventral view **15–16** *Panesthia guizhouensis* sp. n., nymph: **15** paratype, dorsal view **16** same, ventral view. Scale bars = 1.0 cm.

**Figures 17–26.**
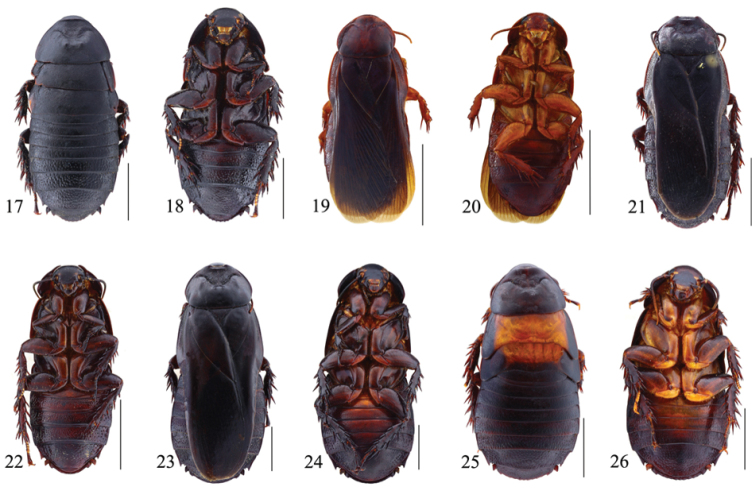
**17–18** *Panesthia strelkovi* Bey-Bienko, 1969, male: **17** dorsal view **18** ventral view **19–20** *Panesthia birmanica* Brunner von Wattenwyl, 1893, male: **19** dorsal view **20** ventral view **21–22** *Panesthia sinuata* Saussure, 1895, male: **21** dorsal view **22** ventral view **23–24** *Panesthia angustipennis cognata* Bey-Bienko, 1969, female: **23** dorsal view **24** ventral view **25–26** *Panesthia angustipennis cognata* Bey-Bienko, 1969, nymph: **25** dorsal view **26** ventral view. Scale bars = 1.0 cm.

##### Panesthia antennata

Taxon classificationAnimaliaBlattodeaBlaberidae

Brunner von Wattenwyl, 1893

Figs 1–8 , 27 –38

Panesthia antennata Brunner von Wattenwyl, 1893: 50; Roth 1979: 61.

###### Description.

**Male.** Body black or dark brown (Figs 1, 5). Face black with brown eyes, ocelli and upper lip yellowish brown. Antennae black, with apical segments pale yellow. Sternites and legs reddish brown or black, tarsal pulvilli pale (Figs 2, 6).

**Figures 27–36.**
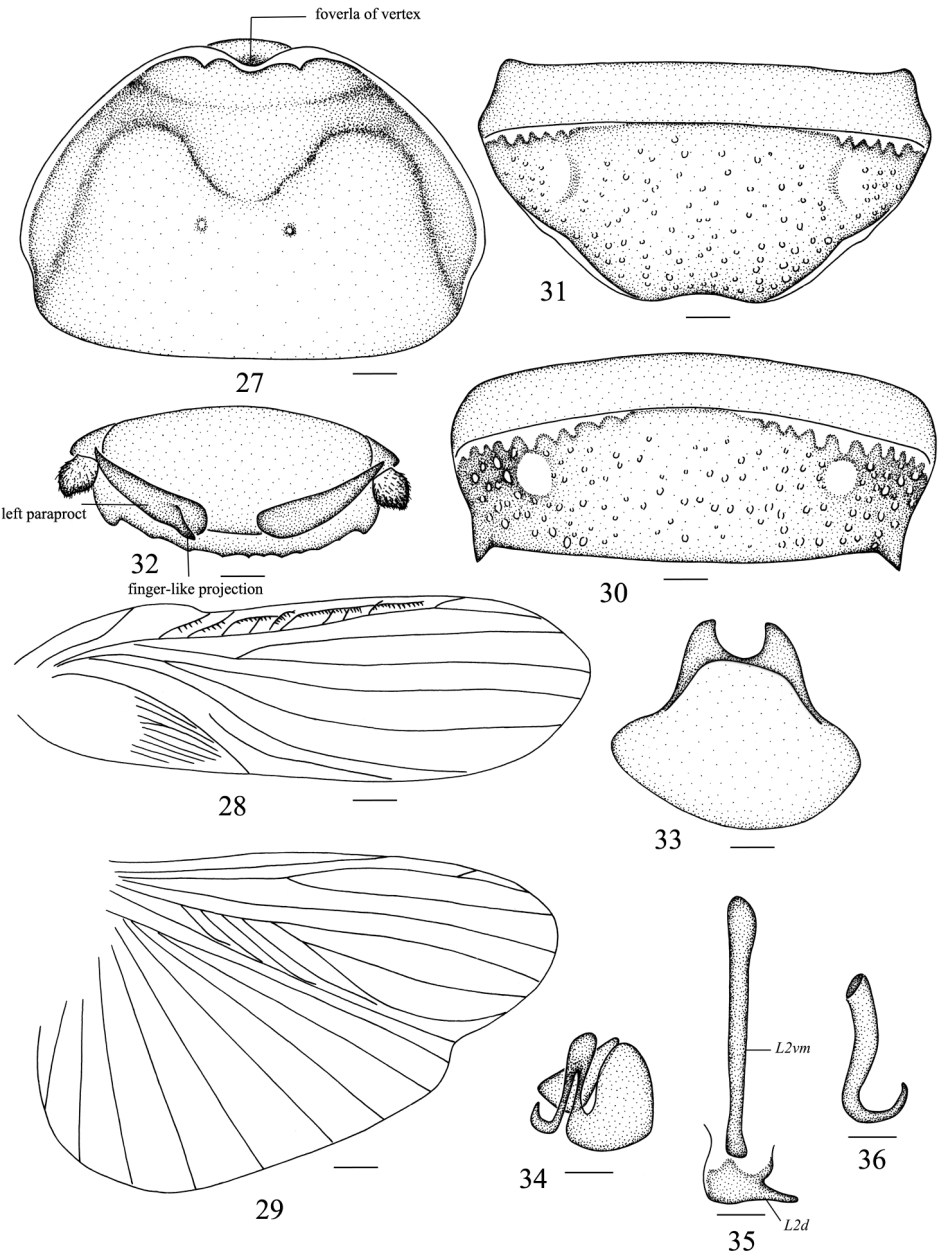
*Panesthia antennata* Brunner von Wattenwyl, 1893 **27** vertex and pronotum **28** tegmen **29** hind wing **30** abdominal tergum 7, dorsal view **31** abdominal sternite 7, ventral view **32** supra-anal fig and paraprocts, ventral view **33** subgenital fig, dorsal view **34** left phallomere (*L1*) **35** median phallomere (*L2vm* and *L2d*) **36** right phallomere (*R2*). Scale bars = 1.0 mm (Figs **27, 30–33**), 2.0 mm (Figs **28–29**), 0.5 mm (Figs **34–36**).

**Figures 37–38.**
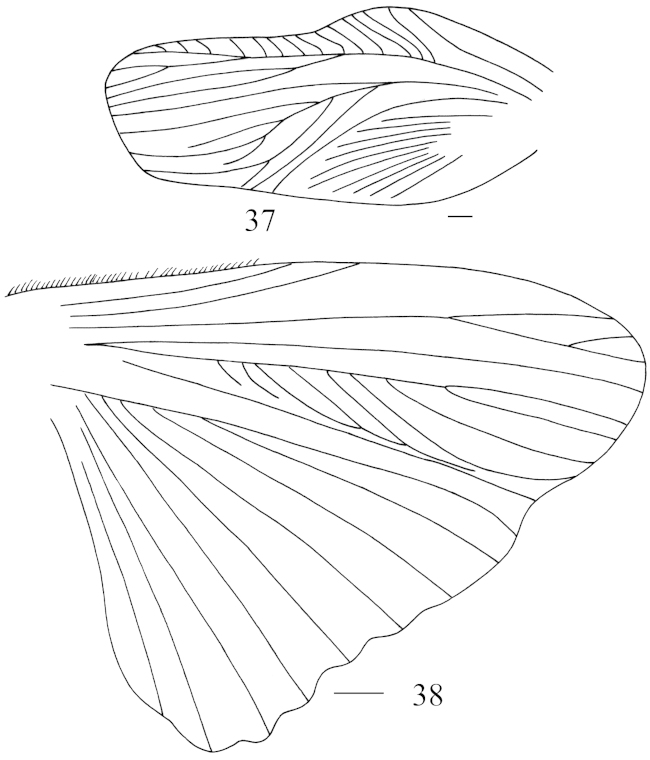
brachypterous form of *Panesthia antennata* Brunner von Wattenwyl, 1893 **37** tegmen **38** hind wing. Scale bars = 1.0 mm.

Vertex punctate, with a small foveola in dorsal view, which is exposed to the excision of pronotum in anterior margin (Fig. 27). Face punctulate, ocelli distinct. Pronotum transverse, anterior margin with a U-shaped excision in the middle, lateral corners of the indentation incrassate and upturned; lateral margins convex and the widest point below the middle; hind margin straight; anterior 1/3 of pronotum weakly depressed and the floor granular, with two rounded grooves, remaining surface punctate and with two tubercles medially (Fig. 27). Tegmina and wings fully developed (Figs 28–29) and reaching or extending beyond the end of abdomen (Fig. 1), sometimes mutilated. In brachypterous form, tegmina and wings reduced (Figs 37–38) with tip just reaching the hind margin of segment two to segment four of abdomen (Fig. 5), sometimes mutilated terminally. Caudal edge of the hind wing wave-shaped (Fig. 38). Anterior ventral margin of front femur with 0–2 spines and distal spine absent, hind margin with a large distal spine. Abdominal tergites densely punctate, the punctations denser caudally, anterolateral corners without holes. Caudal angles of *T6* rounded; lateral margins of *T7* smooth, posteriolateral angles extended caudally and with subacute apex (Fig. 30). Abdominal sternites densely punctate, hind margin of *S7* concave (Fig. 31) and subgenital fig slightly exposed. Supra-anal fig densely punctate, the surface coarser than on abdominal tergites; hind margin crenulate, with 7–10 small teeth in middle, caudal angles small, similar to or slightly bigger than the largest one between them (Fig. 32). Cercus fin-shaped, setaceous ventrally, dorsal surface without setae (Fig. 32). Posterior part of subgenital fig flabellate (Fig. 33).

**Male genitalia.** Genital phallomere *L1* well developed but slightly sclerotized (Fig. 34); *L2vm* rod-like, *L2d* with an elongate projection at the apex (Fig. 35); *R2* developed and hook-shaped (Fig. 36).

**Female.** Similar to male, but hind margin of *S7* rounded. In all specimens observed, the anterior margin of pronotum with an excision. In brachypterous form, tegmina and wings also reduced similar to males.

**Nymph.** Body black and punctate, with two yellowish brown marks on the mesonotum and metanotum without marks (Figs 3–4, 7–8).

**Measurements.** Male, body length: 29.1–32.0 mm; pronotum: length/width: 6.5–6.9/9.6–10.5 mm; width of excision of anterior margin of pronotum: 1.7–2.0 mm; distance between disc tubercles: 1.5–2.0 mm; tegmen: 22.7–26.4 mm. In brachypterous form, body length: 28.5–35.5 mm; pronotum: length/width: 6.8–7.1/9.8–11.3 mm; width of excision of anterior margin of pronotum: 2.0–2.5 mm; distance between disc tubercles: 1.9–2.5 mm; tegmen: 13.5–16.7 mm.

Female, body length: 29.7–32.6 mm; pronotum: length/width: 6.5–7.0/10.1–10.3 mm; width of excision of anterior margin of pronotum: 1.7–2.0 mm; distance between disc tubercles: 1.9–2.0 mm; tegmen: 23.0–26.8 mm. In brachypterous form, body length: 32.0–32.5 mm; pronotum: length/width: 6.1–7.1/9.6–11.1 mm; width of excision of anterior margin of pronotum: 1.6–2.5 mm; distance between disc tubercles: 2.0–2.4 mm; tegmen: 12.6–16.0 mm.

###### Material examined.

Two males, two females and one nymph, China: Yunnan Prov., Tengchong County, Mt. Gaoligong, 9 August 2005, coll. Benyong Mao (HBU); 13 males, six females and 32 nymphs, Yunnan Prov., Tengchong County, Lingjiapu, 13–14 August 2006, coll. Biao Liu (SWFU).

In brachypterous form, one male and one female, China: Yunnan Prov., Tengchong County, Mt. Gaoligong, Baihualing, 30 July 2012, coll. Jishan Xu and Lingxiao Chang (HBU); two males, two females and one nymph, Yunnan Prov., Longling County, Longxin Village, Mt. Hei, 2300m, 23–25 July 2008, coll. Jishan Xu and Zhenhua Gao (HBU); one male and one female, China: Yunnan Prov., Nanjian County, Mt. Wuliang, 8–9 July 2006, coll. Benyong Mao and Juntong Lang (HBU); one male and one nymph, China: Yunnan Prov., Nanjian County, Mt. Wuliang, 17 July 2003, coll. Benyong Mao (HBU); three males and five nymphs, China: Yunnan Prov., Longchuan County, Mt. Ping, 1800m, 6 November 2003, coll. Jinxin Song (SWFU); one male, China: Yunnan Prov., Lijiang, Snow Mt. Yulong, Yuanbinping, 3228m, 31 October 2007, coll. Biao Liu (SWFU); one male and one female, China: Yunnan Prov., Tengchong County, Dahaoping, 2000m, 3 May 2002, coll. Jinxin Song and Yingxian Situ (SWFU); one male, China: Yunnan Prov., Baoshan City, Baihualing, 1980m, 12 April 2002, coll. Yingxian Situ (SWFU); one male and one nymph, Yunnan Prov., Mt. Wuliang, 2000m, 17 July 2003, coll. Benyong Mao (DLU).

###### Distribution.

China (Yunnan); Myanmar.

##### Panesthia stellata concava ssp. n.

Taxon classificationAnimaliaBlattodeaBlaberidae

Figs 9–12 , 39–48

###### Description.

**Male.** Body black (Fig. 9). Eyes, ocelli and upper lip yellowish brown. Antennae black, with terminal segments yellow. Legs black, tarsal pulvilli pale yellowish (Fig. 10).

**Figures 39–48.**
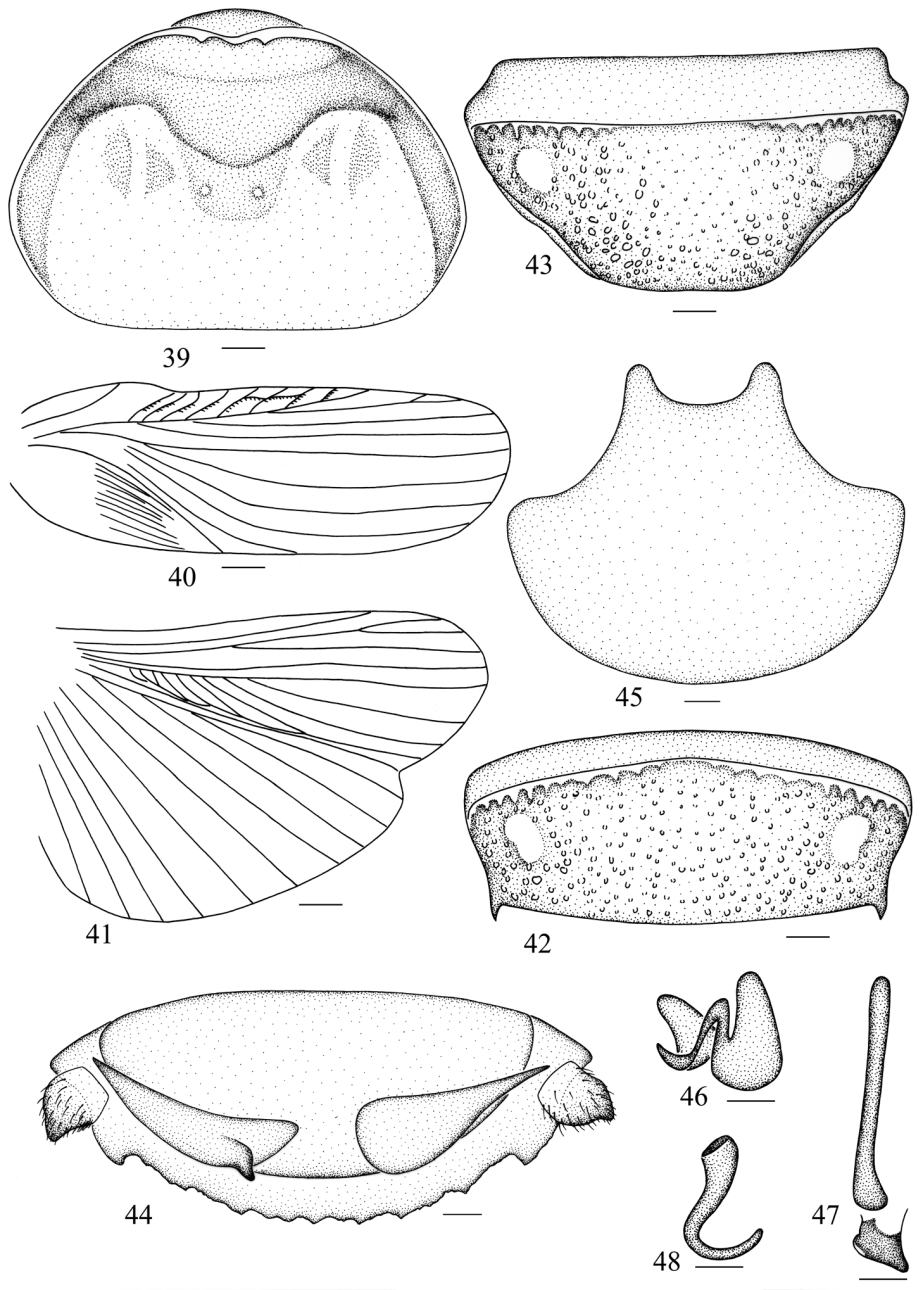
*Panesthia stellata concava* ssp. n. **39** vertex and pronotum **40** tegmen **41** hind wing **42** abdominal tergum 7, dorsal view **43** abdominal sternite 7, ventral view **44** supra-anal fig and paraprocts, ventral view **35** subgenital fig, dorsal view **46** left phallomere (*L1*) **47** median phallomere (*L2vm* and *L2d*) **48** right phallomere (*R2*). Scale bars = 1.0 mm (Figs **39, 42–45**), 2.0 mm (Figs **40–41**), 0.5 mm (Figs **46–48**).

Vertex and face punctate, the former exposed and without foveola (Fig. 39). Ocelli small with indefinite borders. Pronotum transverse, anterior margin concave, thickened and with a V- or U-shaped excision in the middle, the corners of the excavation slightly incrassate and upturned; lateral margins rounded with the widest point behind the midline; hind margin almost straight; anterior 1/3 of pronotum depressed with two arched grooves, the surface granular; posterior half densely punctate, with two middle tubercles (Fig. 39). Tegmina and wings well developed (Figs 40–41), extending to or beyond the end of the abdomen (Fig. 9). Anterior ventral margin of front femur with zero, two or four spines (most commonly two) and a small distal spine, hind margin with a large distal spine. Abdominal tergites densely punctate, the anterolateral corners of tergites without hole; caudal angles of *T6* rounded; lateral margins of *T7* smooth, caudal angles oblique and subacute (Fig. 42). Abdominal sternites densely punctate, hind margin of *S7* truncate and rear edge of subgenital fig exposed (Fig. 43). Supra-anal fig roughened and densely punctate, coarser than abdominal tergites; hind margin with 8–10 subobsolete teeth and with margin uneven; lateral angles larger than the medial tooth (Fig. 44). Cercus without setae dorsally, ventral surface convex with dense hairs (Fig. 44). Anterior margin of subgenital fig concave, anterolateral corners rounded; lateral margins concave (Fig. 45).

**Male genitalia.** Genital phallomere *L1* well developed (Fig. 46); *L2vm* rod-like, *L2d* short and apex round (Fig. 47); *R2* well developed and hook-shaped (Fig. 48).

**Female.** Essentially similar to male, differs with the anterior margin of pronotum weakly concave as well as *S7* with rounded hind margin.

**Nymph.** Body black and punctate, with a broad yellow band on the mesonotum which extends to the middle of the metanotum, hind border of the mark concave (Figs 11–12).

**Measurements.** Male, body length: 27.0–32.5 mm; pronotum: length/width: 5.9–7.3/9.4–11.2 mm; width of excision of anterior margin of pronotum: 1.3–2.0 mm; distance between disc tubercles: 1.6–2.1 mm; tegmen: 22.1–26.7 mm.

Female, body length: 28.5–32.5 mm; pronotum: length/width: 6.5–7.1/10.3–11.0 mm; width of excision of anterior margin of pronotum: 1.0–1.3 mm; distance between disc tubercles: 1.5–2.0 mm; tegmen: 23.8 mm.

###### Material examined.

*Holotype*, male, China: Xizang Prov., Chayu County, Shangchayu Town, 8 August 2013, coll. Xinglong Bai and Junsheng Wang (HBU). *Paratypes*, four males, one female and four nymphs, same data as holotype (HBU); one male, two females and two nymphs, Sichuan Prov., Dege County, Gengqing Town, 3270m, 19 July 2009, coll. Guodong Ren (HBU); one female, China: Xizang Prov., Chayu, 2000m, 24 August 1973, coll. Fusheng Huang (SWU).

###### Remarks.

This subspecies is close to *Panesthia stellata stellata* Saussure, 1895, but can be distinguished by the following characteristics: 1) nymph with broad yellowish band on mesonotum and metanotum, nymph of *Panesthia stellata stellata* with two yellowish markings on mesonotum and without markings on metanotum; 2) anterior margin of pronotum with an excision in both sexes and the corners of the excision upturned in male; anterior margin of pronotum entire or slightly concave in both sexes of *Panesthia stellata stellata*. Feng and Woo (1990) identified the material collected by Fusheng Huang as *Panesthia stellata* Saussure, 1895. But after our critical examination, it should be treated as a new subspecies.

###### Etymology.

The subspecific epithet is derived from the Latin word “*concavus*” which refers to the hind margin of the yellowish mark on nymphs being concave.

##### Panesthia guizhouensis sp. n.

Taxon classificationAnimaliaBlattodeaBlaberidae

http://zoobank.org/D22FF5CD-A07D-43E6-A7E8-0EDBAAEC0CFC

Figs 13–16 , 49–58

###### Description.

**Male.** Body dark brown or black (Fig. 13). Face black, eyes brown or black, ocelli pale yellowish and upper lip yellowish brown. Antennae black, terminal segments light brown. Abdominal sternites reddish brown with the middle of anterior three sternites brown (Fig. 14). Legs reddish brown with coxae and trochanter brown, tarsal pulvilli pale (Fig. 14).

**Figures 49–58.**
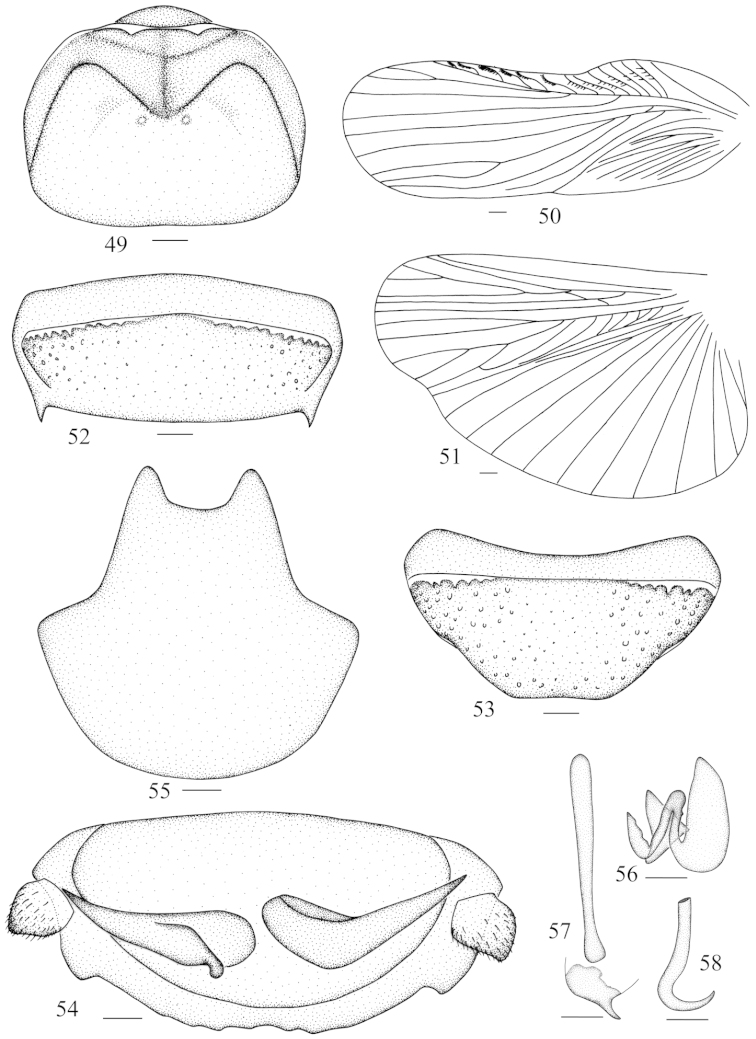
*Panesthia guizhouensis* sp. n. **49** vertex and pronotum **50** tegmen **51** hind wing **52** abdominal tergum 7, dorsal view **53** abdominal sternite 7, ventral view **54** supra-anal fig and paraprocts, ventral view **55** subgenital fig, dorsal view **56** left phallomere (*L1*) **57** median phallomere (*L2vm* and *L2d*) **58** right phallomere (*R2*). Scale bars = 1.0 mm (Figs **49–55**), 0.5 mm (Figs **56–58**).

Vertex slightly punctate, exposed (Fig. 49). Face punctulate, ocelli small, round. Pronotum transverse ovate and flat; anterior margin convex or straight, center weakly concave, lateral corners of the indentation slightly incrassate and upturned; lateral margins convex and widest at or before the midline; hind margin straight; anterior 1/3 of pronotum shallowly depressed and delineated by two grooves, the surface sparsely granular; posterior half flattened and punctate densely, with two small disc tubercles (Fig. 49). Tegmina and wings well developed (Figs 50–51), extending to or surpassing the tip of abdomen (Fig. 13), few reaching to the hind margin of 6^th^ tergite, sometimes mutilated terminally. Anterior ventral margin of front femur with 0–1 spine and with a small distal spine, hind margin with a large distal spine. Abdominal tergites punctate, with punctations denser caudally, anterolateral corners without holes. Caudal angles of 6^th^ tergite weakly extended; lateral margins of 7^th^ tergite smooth, caudal angles acute and directed caudally (Fig. 52). Abdominal sternites punctate, hind margin of the 7^th^ sternite weakly concave and subgenital fig exposed (Fig. 53). Supra-anal fig punctate densely, hairless, hind margin smooth or slightly concave in the middle in dorsal view, with 5–7 small teeth medially or smooth in ventral view, caudal angles small (Fig. 55). Cercus fin-shaped with acute apex, dorsal surface without setae and hairy ventrally (Fig. 54). Hind margin of subgenital fig rounded (Fig. 55).

**Male genitalia.** Genital phallomere well developed, *L1* well sclerotized (Fig. 56); *L2vm* rod-like, *L2d* elongate (Fig. 57); *R2* hook-shaped (Fig. 58).

**Female.** Differs from male as follows: the anterior margin of pronotum weakly concave and the corners not upturned; the tubercles on surface smaller than in male. Hind margin of *S7* rounded.

**Nymph.** Body black and punctuate, with two yellowish brown marks on the mesonotum and metanotum without marks (Figs 15–16).

**Measurements.** Male, body length: 24.5–27.0 mm; pronotum: length/width: 4.2–5.2/ 7.9–9.0 mm; width of excision of anterior margin of pronotum: 1.5–1.8 mm; distance between disc tubercles: 1.1–1.5 mm; tegmen: 22.0–25.6 mm.

Female, body length: 26.8–31.5 mm; pronotum: length/width: 4.5–5.0/8.5–9.0 mm; width of excision of anterior margin of pronotum: 1.1–1.3 mm; distance between disc tubercles: 1.3–1.5 mm; tegmen: 21.2–23.2 mm.

###### Material examined.

*Holotype*, male, China: Guizhou Prov., Zunyi City, Suiyang County, Kuankuoshui Natural Reserve, 14 August 2010, coll. Keliang Wu (SWU). *Paratypes*, five males, two females and five nymphs, same data as holotype (SWU); four males, 12 females and 13 nymphs, Guizhou Prov., Zunyi City, Suiyang County, Kuankuoshui Natural Reserve, 1–2 August 2013, coll. Xiudan Wang and Yuhong Zheng (SWU).

###### Remarks.

This species is similar to *Panesthia angustipennis spadica*, but can be distinguished by the following characteristics: 1) anterior margin of pronotum weakly concave in male, and without mesal elevation, male of latter with anterior margin broadly excised and with mesal elevation; 2) body length < 30 mm in most, or few surpassing 30 mm, body length of *Panesthia angustipennis spadica* > 30 mm.

###### Etymology.

The specific epithet is named after the locality of the holotype, Guizhou Province.

##### Panesthia strelkovi

Taxon classificationAnimaliaBlattodeaBlaberidae

Bey-Bienko, 1969

Figs 17–18 , 59–67

Panesthia strelkovi Bey-Bienko, 1969: 834; Roth 1979: 104; Feng and Woo 1990: 216.

###### Description.

**Male.** Body black (Fig. 17). Face black, eyes and ocelli brown, upper lip yellowish brown, antennae black with brownish apex. Legs dark brown, coxae and trochanter reddish brown (Fig. 18).

**Figures 59–67.**
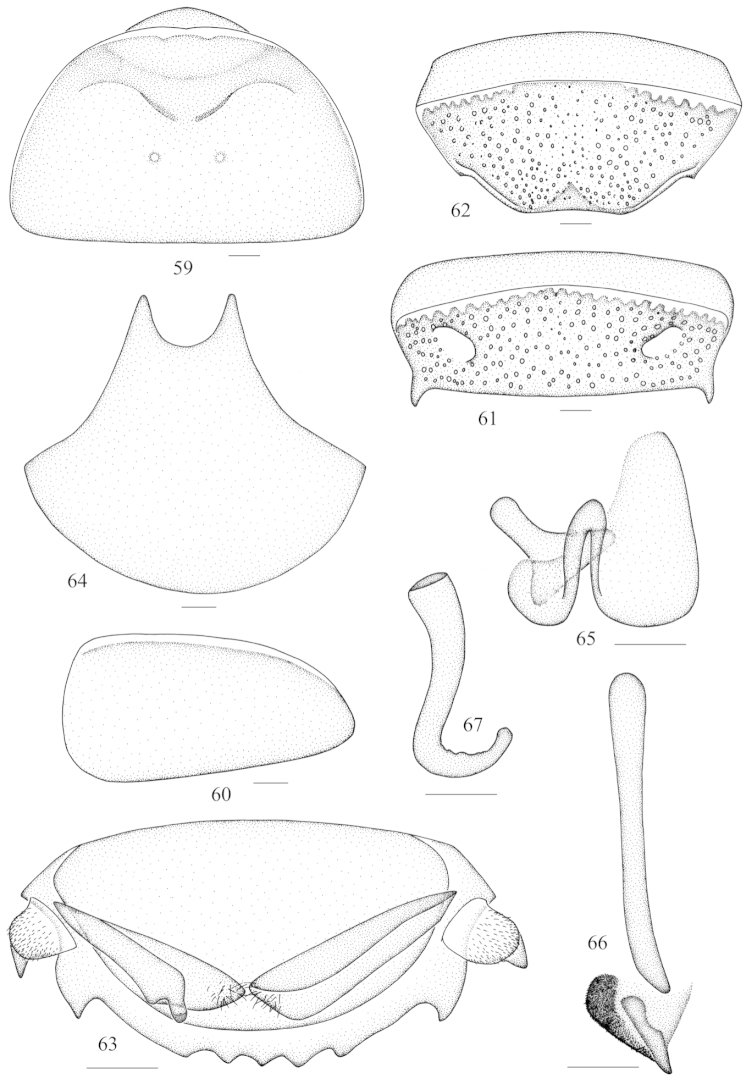
*Panesthia strelkovi* Bey-Bienko, 1969 **59** vertex and pronotum **60** tegmen **61** abdominal tergum 7, dorsal view **62** abdominal sternite 7, ventral view **63** supra-anal fig and paraprocts, ventral view **64** subgenital fig, dorsal view **65** left phallomere (*L1*) **66** median phallomere (*L2vm* and *L2d*) **67** right phallomere (*R2*). Scale bars = 1.0 mm (Figs **59, 61–62**), 0.5 mm (Figs **60, 63–67**).

Face punctulate, with weak ocelli; vertex slightly exposed (Fig. 59). Pronotum nearly semicircular, the widest near the truncate hind margin (Fig. 59). Anterior margin convex and incrassate, slightly concave in the middle, with two upturned tubercles (Fig. 59). Anterior half of pronotum depressed, the surface granular; posterior part punctate and elevated, with two tubercles on disc (Fig. 59). Tegmina reduced to sclerotized blades (Fig. 60) which are far separated on the lateral sides of the mesonotum respectively, with apex slightly surpassing hind margin of mesonotum; wings absent (Fig. 17). Anterior ventral margin of front femur with 2–3 spines and with one small distal spine, hind margin with one large distal spine. Abdominal tergites densely punctate, with punctures denser and larger caudally; lateral margins smooth; anterolateral corners without holes. *T6* with small spine in the each hind angle, *T7* with relatively large posteriolateral angles, with apexes acute and directed to the terminus (Fig. 61). Abdominal sternites equally punctate. Lateral margins of *S7* oblique and protruded medially; hind margin straight (Fig. 62) and subgenital fig marginally exposed. The surface of supra-anal fig uniformly covered with large round punctations; hind margin with 5–7 small subacute teeth medially; the lateral teeth with acute apexes are larger than the teeth between them (Fig. 63). Cercus fin-shaped and pointed apically, swollen and hirsute in venter, but hairless dorsally (Fig. 63). Anterior angles of subgenital fig tapering to subacute apexes (Fig. 64).

**Male genitalia.** *L1* well developed (Fig. 65); *L2vm* stick-like and *L2d* slightly sclerotized, which is hairy in left side of dorsal view (Fig. 66); R2 hook-shaped with apex rounded (Fig. 67).

**Measurements.** Male, body length: 29.0–37.0 mm; pronotum: length/width: 7.2–8.2/10.0–13.0 mm; width of excision of anterior margin of pronotum: 1.0–1.7 mm; distance between disc tubercles: 2.0–2.5 mm; tegmen: 3.6–5.5 mm.

###### Material examined.

Two males, China: Hainan Prov., Mt. Diaoluo, 12 May 1965, coll. Sikong Liu; one male, China: Hainan Prov., Mt. Jianfengling, 17 April 1982, coll. Zhiqin Chen. (SWU)

###### Distribution.

China (Hainan).

##### Panesthia birmanica

Taxon classificationAnimaliaBlattodeaBlaberidae

Brunner von Wattenwyl, 1893

Figs 19–20 , 68–73

Panesthia birmanica Brunner, 1893: 54; Roth 1979: 67; Feng and Woo 1990: 213.

###### Remarks.

**Figures 68–73.**
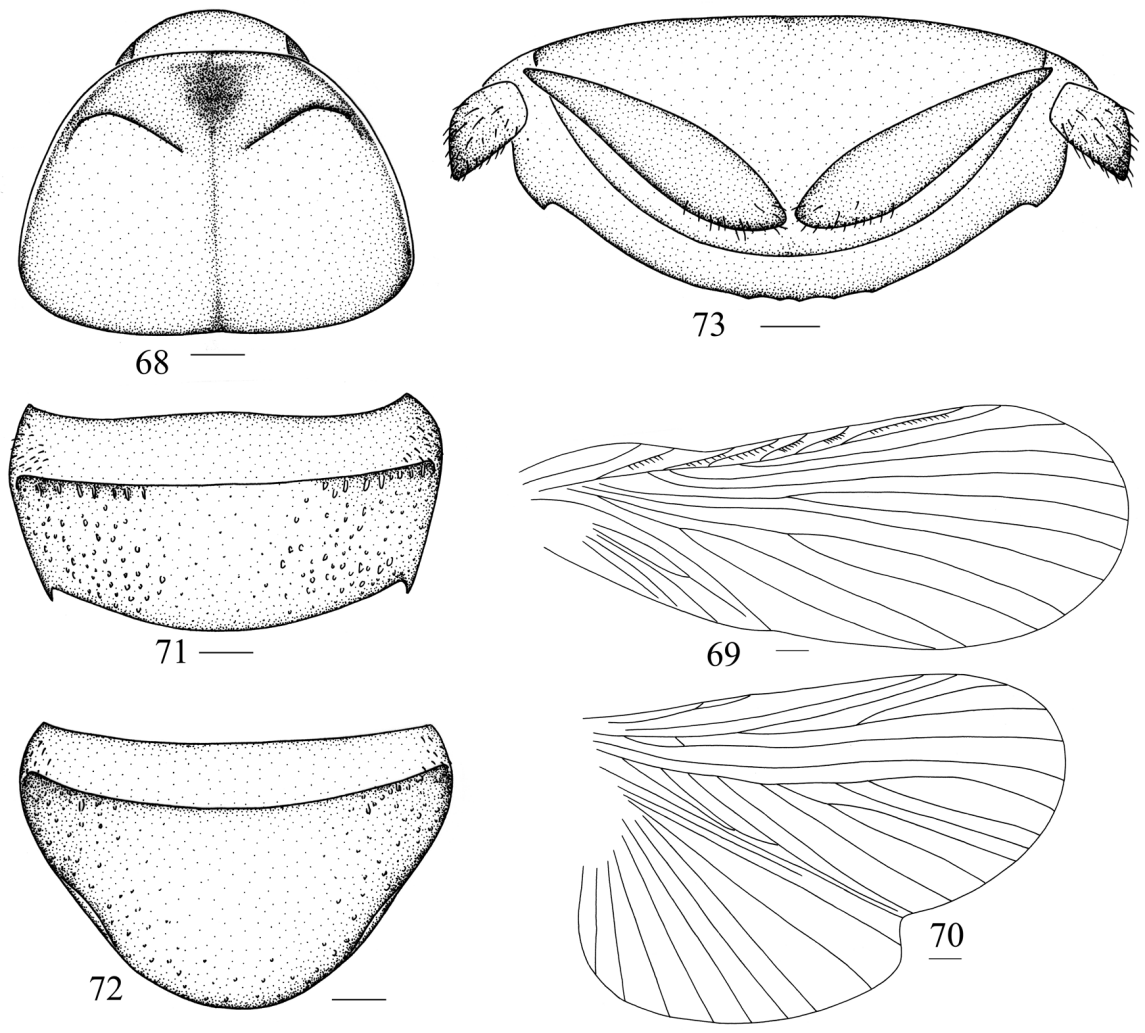
*Panesthia birmanica* Brunner von Wattenwyl, 1893, female **68** vertex and pronotum **69** tegmen **70** abdominal tergum 7, dorsal view **71** hind wing **72** abdominal sternite 7, ventral view **73** supra-anal fig and paraprocts, ventral view. Scale bars = 1.0 mm (Figs **68–72**), 0.5 mm (Fig. **73**).

This species is distinguished from other species by its small body length, ranging from 22.5 mm to 28 mm; and also by pronotum virtually flattened, anterior margin slightly thickened, entire or weakly indented in male, entire and not thickened in female (Fig. 68); as well as the caudal angles of *T7* acute and posteriorly directed (Fig. 71); hind margin of supra-anal fig smooth or slightly crenulate, with the lateral teeth acute in apexes (Fig. 73); anterior ventral margin of front femur with 0–3 spines and with small distal spine, and posterior ventral margin with a large distal spine. The caudal angles of *T6* sometimes with an acute spine separately may confuse this species with species in the genus *Miopanesthia*, but can be distinguished by the shorter hind metatarsus, as stated in the genus remarks. Roth (1979) determined that the tegmina and wings were polymorphic in this species since the specimens he examined had both the macropterous and brachypterous forms. The tegmina and wings are fully developed (Figs 19, 69–70) or mutilated on all material of this species we examined.

###### Material examined.

One female, China: Hainan Prov., Mt. Jianfengling, Tianchi, 6 July 1981, collector unknown; one female, China: Hainan Prov., Changjiang, Mt. Bawangling, 8–13 July 2006, coll. Jiliang Wang and Chao Gao; three females, China: Yunnan Prov., Xishuangbanna, Dadugang, 27 April 2014, Xinran Li. (SWU).

###### Distribution.

China (Hainan, Yunnan); India; Myanmar; Vietnam; Thailand.

##### Panesthia sinuata

Taxon classificationAnimaliaBlattodeaBlaberidae

Saussure, 1895

Figs 21–22 , 74–83

Panesthia sinuata Saussure, 1895: 318; Roth 1979: 55; Feng and Woo 1990: 216.

###### Remarks.

**Figures 74–83.**
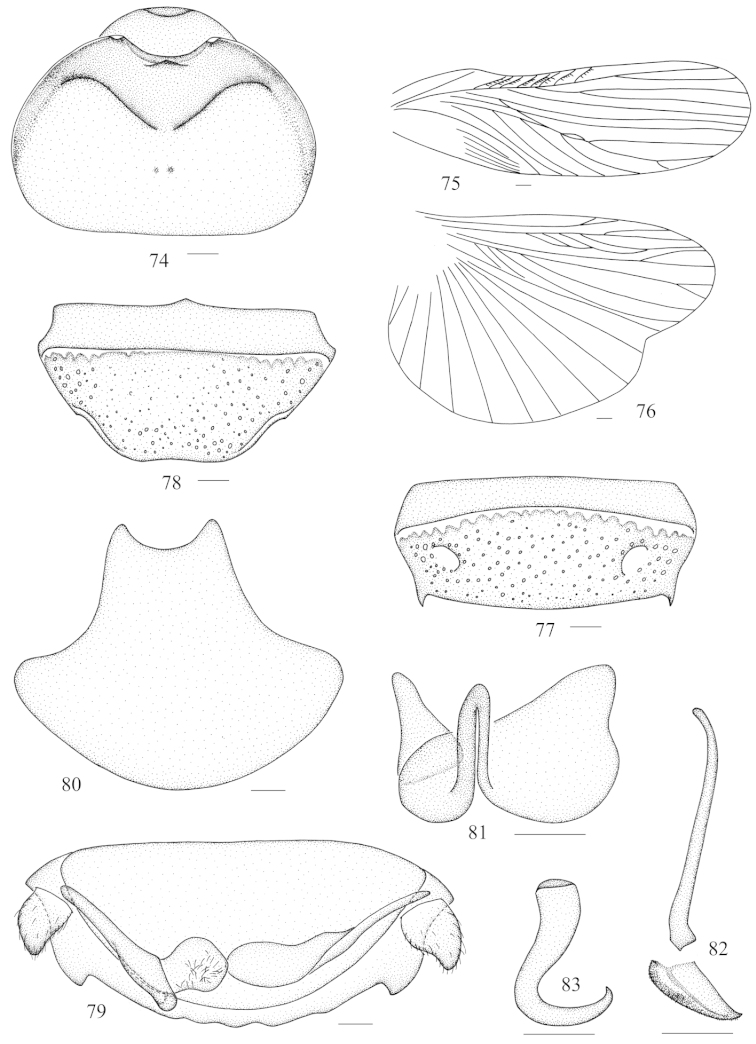
*Panesthia sinuata* Saussure, 1895 **74** vertex and pronotum **75** tegmen **76** hind wing **77** abdominal tergum 7, dorsal view **78** abdominal sternite 7, ventral view **79** supra-anal fig and paraprocts, ventral view **80** subgenital fig, dorsal view **81** left phallomere (*L1*) **82** median phallomere (*L2vm* and *L2d*) **83** right phallomere (*R2*). Scale bars = 1.0 mm (Figs **74–78**), 0.5 mm (Figs **79–83**).

This species is similar to *Panesthia angustipennis spadica*, but can be distinguished by body length (19–29 mm), which is commonly smaller than the latter (34–42 mm). In male of *Panesthia sinuata*, the anterior margin of pronotum is broadly concave and the corners of the indentation upturned (Fig. 74), while the latter is merely concave and without corners in small individuals about 34 mm. The anteroventral margin of front femur often bears 0–1 spine in *Panesthia sinuata*, but more than two spines in *Panesthia angustipennis spadica*. Roth (1979) described this species as similar to *Panesthia antennata*, but the male of the latter had no mesal elevation of the anterior margin of pronotum.

###### Material examined.

Two males and one female, China: Yunnan Prov., Xishuangbanna, Menghai, 1200–1600m, 26 July 1958, coll. Shuyong Wang; one male, China: Yunnan Prov., Xishuangbanna, 1050–1080m, 9 June 1958, coll. Fuji Pu. (SWU)

###### Distribution.

China (Yunnan, Guangdong); Vietnam; Laos; Malaysia.

##### Panesthia angustipennis cognata

Taxon classificationAnimaliaBlattodeaBlaberidae

Bey-Bienko, 1969

Figs 23–26 , 84–93

Panesthia cognata Bey-Bienko, 1969: 833; Feng and Woo 1990: 216.
Panesthia angustipennis cognata , Roth 1979: 42.

###### Remarks.

This subspecies resembles *Panesthia angustipennis spadica*, and it is difficult to distinguish adults. Roth (1979) stated *Panesthia angustipennis spadica* had a deflexed hind margin of the supra-anal fig and appeared to be entire dorsally, not deflexed and with teeth visible dorsally in *Panesthia angustipennis cognata*. Despite this character being variable, some of the populations were sufficiently different, and distinct on average. It is significant that *L2d* of median phallomere in *Panesthia angustipennis cognata* is short with variable apex, but elongate and tapering to a round apex in *Panesthia angustipennis spadica*. Nymphs of *Panesthia angustipennis cognata* have a broadly yellowish marking on mesonotum and metanotum (Fig. 25) while *Panesthia angustipennis spadica* is with or without two markings on mesonotum, and without any markings on metanotum.

**Figures 84–93.**
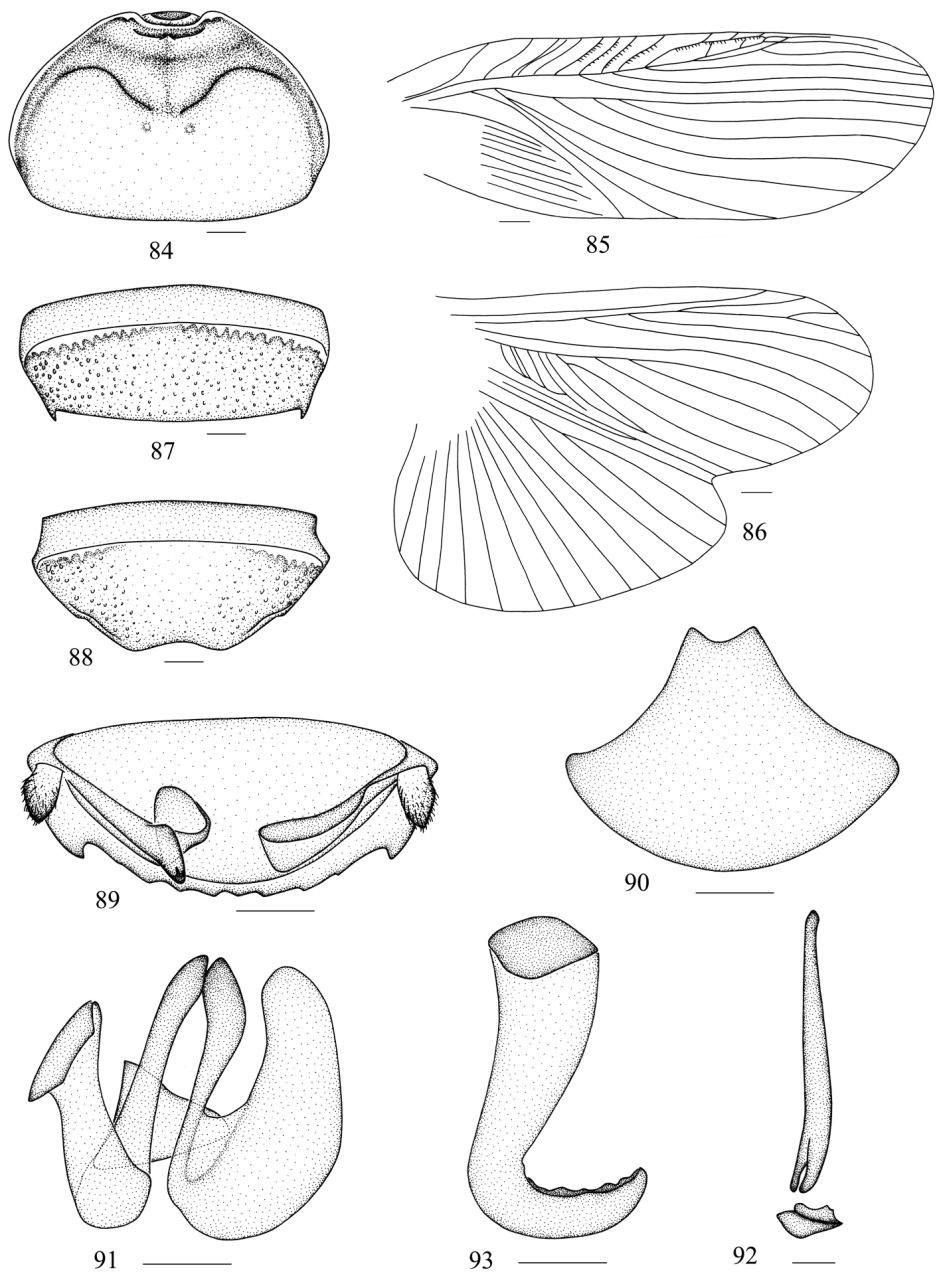
*Panesthia angustipennis cognata* Bey-Bienko, 1969 **84** vertex and pronotum **85** tegmen **86** hind wing **87** abdominal tergum 7, dorsal view **88** abdominal sternite 7, ventral view **89** supra-anal fig and paraprocts, ventral view **90** subgenital fig, dorsal view **91** left phallomere (*L1*) **92** median phallomere (*L2vm* and *L2d*) **93** right phallomere (*R2*). Scale bars = 2.0 mm (Figs **84–90**), 0.5 mm (Figs **91–93**).

###### Material examined.

One nymph, China: Hainan Prov., Mt. Jianfengling, 29 June 1981, coll. Kegang Hu; one nymph, China: Hainan Prov., Ledong County, 25 December 1963, coll. Yuliang Luo; one nymph, China: Hainan Prov., Mt. Jianfengling, Tianchi, 24 February 1982, Ruilin Pan; one female, China: Hainan Prov., Mt. Jianfengling, 24 November 1981, Ruilin Pan; one nymph, China: Hainan Prov., Mt. Jianfengling, 8 July 1981, collector unknown; one nymph, China: Hainan Prov., Mt. Jianfengling, Tianchi, 8–10 May 1964, coll. Hui Ren; one nymph, China: Hainan Prov., Mt. Jianfengling, 11 May 1981, collector unknown; one female, China: Hainan Prov., Ledong County, Mt. Jianfengling, 1050m, 6–7 December 2007, coll. Weiwei Zhang; one female, China: Hainan Prov., Mt. Jianfengling, Tianchi, 26 July 1983, coll. Lishen Hua; one nymph, China: Hainan Prov., Wanning City, Xinglong, 23 February 1964, coll. Sikong Liu; one female, China: Hainan Prov., Mt. Jianfengling, 9 May 1964, coll. Sikong Liu; one nymph, China: Yunnan Prov., Menglun, 1 August 2009, coll. Zongqing Wang; one nymph, China: Yunnan Prov., Yingjiang County, Tongbiguan, 1450m, 30 July 2009, coll. Benyong Mao; one male, China: Guizhou Prov., Liping County, Mt. Taiping, 27 July 2009, coll. Yang; two males, one female and one nymph, China: Xizang Prov., Motuo, 1100m, 5 January 1983, coll. Yinheng Han; one female, China: Xizang Prov., Motuo, 700-1050m, 23 June 1983, coll. Yinheng Han; one male, China: Xizang Prov., Chayu County, Songgu, 23 July 1972, coll. Fusheng Huang. (SWU). One male, one female and one nymph, China: Guangxi Prov., Hechi City, Tian'e County, 14–19 September 2002, coll. Ming Bai. (HBU)

###### Distribution.

China (Hainan, Guangxi, Guizhou, Yunnan, Xizang); India; Sikkim; Myanmar; Vietnam; Laos; Thailand.

## Discussion

Gregarious, xylophagous cockroaches of the blaberid genus *Panesthia* exhibit little variation in morphology. They have dark, hard, rigid and pitted exoskeletons. Body sizes range from 15 mm to over 50 mm. They usually live in decaying wood, fallen leaves, rubbish, cracks in rocks, or in some kind of debris, and feed on wood (Feng and Woo 1990). Members of *Panesthia cribrata* live not only inside decaying logs but also under logs (Rugg and Rose 1989). During our collection in Guizhou Province in August, 2013, we obtained a colony of *Panesthia guizhouensis* sp. n. from rotten wood near a large pool, comprised of 52 adults and at least 60 nymphs of different instars. When the wood was split, all of them fled away quickly (Wang X.D., pers. obs.).

Nymphs of *Panesthia angustipennis spadica* have two morphs. One has a pair of large reddish spots on mesonotum (Roth 1979: Fig. 20B) and is from Taiwan, China and Yayeyama Island, Japan. The other is uniformly colored without spots on mesonotum (Roth 1979: Fig. 20A) and is from Taiwan and Japan except Yayeyama Island. Asahina (1988) separated *Panesthia angustipennis yayeyamensis* from *Panesthia angustipennis spadica* in view of the nymph having a reddish marked mesonotum. Maekawa et al. (1999) analyzed the molecular phylogenetic relationships of *Salganea* and *Panesthia* based on the COII gene. *Panesthia angustipennis yayeyamensis* formed a monophyletic clade, which was embedded in the clade of *Panesthia angustipennis spadica*; this suggests *Panesthia angustipennis yayeyamensis* should be returned to *Panesthia angustipennis spadica*. After examination of a large quantity of nymphs of *Panesthia angustipennis*, we think it is not reasonable and adequate to accurately distinguish subspecies only according to the difference in marks of the nymph. For example, there are also different nymph morphs in *Panesthia angustipennis angustipennis* (Roth 1979: Figs 2B–K). Therefore, we hereby agree with the decision by Maekawa et al. (1999) that *Panesthia angustipennis yayeyamensis* should be treated as the synonym of *Panesthia angustipennis spadica*.

*Panesthia* is the only genus of the subfamily Panesthiinae in which several species and subspecies have tegmina and wings which are fully developed or variably reduced (Roth 1982), i.e., include wing polymorphic species. There are five species and four subspecies of *Panesthia* with variable reduction of tegmina and wings in both sexes (Roth 1982). From a *Panesthia antennata*, which was recorded with mutilated tegmina and wings, Roth (1979) inferred that a developed winged morph may have existed. But after checking specimens collected from Yunnan Province, we have identified and discovered 15 males and eight females of *Panesthia antennata* with tegmina and wings about or beyond the end of abdomen, and 11 males and five females with tegmina and wings apparently short, only reaching between the second segment and the fourth segment of the abdomen. Given the above, *Panesthia antennata* can also be treated as a wing polymorphic species.

The reason for wing polymorphism in cockroaches is still unknown. Roth (1977, 1979) observed that some species of *Panesthia* with reduced-wing forms were not commonly collected. We record 16 adult specimens of *Panesthia antennata* in brachypterous form collected (39 specimens in all). To be unambiguous, this brachypterous form does not just occur by accident or gene mutation in rare specimens, but rather in large numbers. Species with both macropterous and brachypterous forms possess a higher fitness (Roff, 1986) and this seems reasonable in *Panesthia* as well. However further investigation will be required to confirm this reasoning.

## Supplementary Material

XML Treatment for Panesthia

XML Treatment for Panesthia antennata

XML Treatment for Panesthia stellata concava

XML Treatment for Panesthia guizhouensis

XML Treatment for Panesthia strelkovi

XML Treatment for Panesthia birmanica

XML Treatment for Panesthia sinuata

XML Treatment for Panesthia angustipennis cognata
